# Total Knee Arthroplasty Designed to Accommodate the Presence or Absence of the Posterior Cruciate Ligament

**DOI:** 10.1155/2014/178156

**Published:** 2014-10-08

**Authors:** Melinda K. Harman, Stephanie J. Bonin, Chris J. Leslie, Scott A. Banks, W. Andrew Hodge

**Affiliations:** ^1^Department of Bioengineering, 301 Rhodes Engineering Research Center, Clemson University, Clemson, SC 29634-0905, USA; ^2^MEA Forensic Engineers & Scientists, 23281 Vista Grande Drive, Laguna Hills, CA 92653, USA; ^3^Leslie Orthopaedics & Sports Medicine, 226 East U.S. Highway 54, Camdenton, MO 65020, USA; ^4^Department Mechanical & Aerospace Engineering, MAEA 318, University of Florida, P.O. Box 116250, Gainesville, FL 32611, USA; ^5^Department of Orthopaedics, Eastern Maine Medical Center, 489 State Street, Bangor, ME 04401, USA

## Abstract

Evidence for selecting the same total knee arthroplasty prosthesis whether the posterior cruciate ligament (PCL) is retained or resected is rarely documented. This study reports prospective midterm clinical, radiographic, and functional outcomes of a fixed-bearing design implanted using two different surgical techniques. The PCL was completely retained in 116 knees and completely resected in 43 knees. For the entire cohort, clinical knee (96 ± 7) and function (92 ± 13) scores and radiographic outcomes were good to excellent for 84% of patients after 5–10 years in vivo. Range of motion averaged 124° ± 9°, with 126 knees exhibiting ≥120° flexion. Small differences in average knee flexion and function scores were noted, with the PCL-resected group exhibiting an average of 5° more flexion but an average function score that was 7 points lower compared to the PCL-retained group. Fluoroscopic analysis of 33 knees revealed stable tibiofemoral translations. This study demonstrates that a TKA articular design with progressive congruency in the lateral compartment can provide for femoral condyle rollback in maximal flexion activities and achieve good clinical and functional performance in patients with PCL-retained and PCL-resected TKA. This TKA design proved suitable for use with either surgical technique, providing surgeons with the choice of maintaining or sacrificing the PCL.

## 1. Introduction

Contemporary total knee arthroplasty (TKA) provides reliable pain relief and restoration of moderate function for patients suffering from severe joint degeneration. Outcomes are typically very good [1, 2], but many times patients do not regain a normal range of motion and strength. In particular, TKA designs that allow excessive anterior-posterior (AP) translation of the femur with respect to the tibia (knee instability) exhibit reduced knee flexion, diminished functional strength, and unfavorable conditions for bearing surface wear [3–13]. Excessive AP motion in well-aligned prostheses occurs with the femur sliding anterior on the tibia in flexion and posterior in extension, resulting in limited femoral rollback and the potential of bony impingement between the femur bone and posterior rim of the tibial insert [5, 14–16]. This instability appears to result from the loss of the knee's natural intrinsic stabilizing structures after TKA, including one or both of the cruciate ligaments and the menisci [17]. Therefore, controlling AP translation of the femur, in the presence or absence of the posterior cruciate ligament (PCL), is often cited as a means for achieving optimal function in modern TKA designs [5, 16, 18–26].

Traditionally, orthopaedic surgeons have been trained to execute surgical techniques that depend on the integrity of the posterior cruciate ligament by utilizing one of two basic TKA designs, the cruciate-retaining type (CR) or the posterior-stabilized type (PS). However, joint laxity can vary widely after TKA due to common variations in these surgical techniques, including whether the PCL is preserved with a tibial bone block at the insertion site, recessed to the level of the tibial bone cut, or completely resected [27–29]. TKA designs incorporate different articular constraints in order to accommodate such variations [30]. While it previously has been suggested that some TKA designs are suitable for use regardless of whether the PCL is retained or resected [31], the clinical outcomes and biomechanical considerations of such designs rarely have been considered [28, 29]. Moreover, it remains unclear precisely how much conformity is required for successful TKA [30].

**Figure 1:**
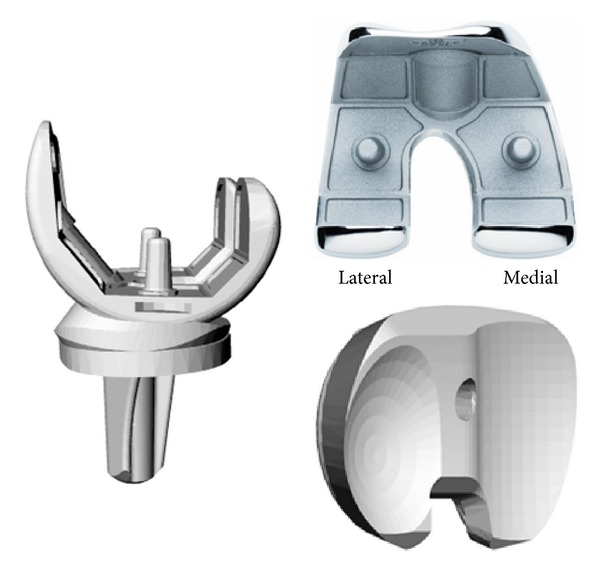
The 3D Knee is a fixed-bearing total knee prosthesis suitable for use in PCL-retained or PCL-resected TKA.

**Figure 2:**
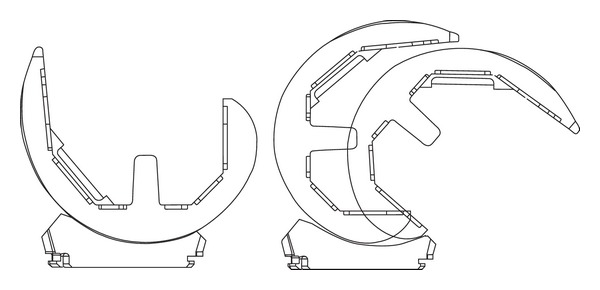
The 3D Knee fixed-bearing TKA design incorporates a hemispherical lateral condyle and tibial articulation to provide definitive AP translational control while providing for proper axial rotation. The asymmetric femoral component incorporates a constant sagittal radius from −15° to 80° while providing progressively decreasing articular constraint with higher flexion to allow femoral condyle rollback. The posterior condyles are shaped to provide maximum posterior condylar offset late in the flexion arc.

The current study was initiated in order to gather evidence documenting the performance of a new TKA articular design concept that incorporates progressive congruency in the lateral compartment (Figures 1 and 2). This design does not require use of a femoral cam and tibial post articulation typical of PS designs, helping to conserve femoral bone and avoiding complications associated with wear of the tibial post [32, 33]. It is implanted using a PCL-retaining surgical technique, which avoids widening of the flexion gap known to occur with PCL resection [34]. Moreover, the highly congruent lateral femoral condyle and widened medial condyle that provide for large contact areas help to optimize load distribution and lower contact stresses compared to other fixed-bearing, CR, and PS TKA designs [35]. The purpose of this study was to characterize the midterm clinical and functional outcomes of a TKA designed to improve knee function by providing more intrinsic AP stability after arthroplasty. Patient populations operated by two surgeons, one utilizing a PCL-retaining surgical technique and the other utilizing a technique with complete PCL resection, were compared.

## 2. Materials and Methods

Two clinical sites in the United States participated in this prospective IRB-approved study to record clinical results of a fixed-bearing TKA design (3D Knee, DJO Surgical, Austin, TX) implanted using two different surgical techniques; either the PCL was retained with a bone block or the PCL was completely resected. Patients with surgery dates between January, 2002 and December, 2006, were identified from TKA surgical databases provided by the two surgeons, providing for evaluation at a minimum follow-up of five years. Inclusion criteria for this study required that patients be older than 21 years, demonstrate severe arthritis with bone to bone disease in at least one knee compartment, have varus or valgus deformity not exceeding 15 degrees, not have had prior knee sepsis, and not have undergone prior knee arthoplasty for any reason. Patients were required to provide informed consent and to be willing to travel to the clinical office to undergo physical evaluation and a radiological exam. Eligible patients were contacted by telephone to schedule clinical visits and were enrolled sequentially until the enrollment target of 150 TKA was achieved.

During the four-year operative period, a total of 251 TKA were implanted by the two participating surgeons using the same implant design. A total of 134 patients (159 TKA) met the inclusion criteria and were willing to participate in the study, including 116 of 193 (60%) TKA implanted by a surgeon who preserved the PCL with a bone block and 43 of 58 (74%) TKA implanted by a surgeon who completely resected the PCL. There were 84 female patients (98 TKA) and 50 male patients (61 TKA) with an average age of 69 ± 9 (range, 33 to 89) years at the time of index TKA. Average duration of function for all TKA was 8.0 ± 1.1 (range, 5.6 to 10.3) years.

Surgical exposure for both surgeons at index TKA was a mid-vastus approach through a standard midline skin incision. Handling of the PCL followed the surgical techniques routinely used by each participating surgeon. Preservation of the PCL was accomplished with a bone block surgical technique, whereas resection of the PCL was accomplished through complete ligament removal and insertion of a Hohmann retractor for anterior tibial dislocation. All knees in both groups were implanted with the same fixed-bearing TKA design (3D Knee), including patellar resurfacing and cement fixation of all three components. All modular tibial inserts and domed patellar components were machined from compression molded polyethylene and were sterilized using gamma radiation and packaged in nitrogen gas.

Patients were followed up prospectively to record clinical and radiological outcomes. The physical evaluation included measurement of maximum knee flexion using a hand-held goniometer and assessment of clinical outcome using the Knee Society Score (KSS knee and function scores) [36]. The radiological exam included acquisition of standard-length radiographs with the patients in a standing, weight-bearing posture, which were obtained at the immediate postoperative and annual intervals. Radiolucent lines were evaluated in predefined zones about the fixation interfaces of the femoral, tibial, and patellar components [37] and graded as none, narrow (1-2 mm), and wide (>3 mm) based on the total width of any observed radiolucent line.

**Figure 3:**
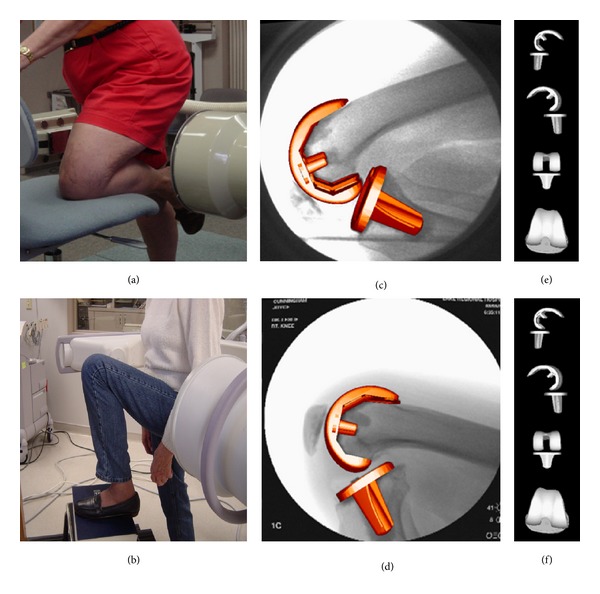
In vivo sagittal plane fluoroscopic images acquired during kneeling and lunge activities (a, b) were analyzed using a shape-matching procedure for fitting the prosthesis surface models to the image silhouettes (c, d) and calculating the position and orientation of the femoral component relative to the tibial component (e, f).

In addition to the clinical and radiological outcomes described above, some patients were requested to participate in a quantitative analysis of two deep flexion activities in the early follow-up period (3 to 13 months), including 20 patients from the PCL-retained group and 13 patients from the PCL-resected group. Similar to previous fluoroscopy studies [4, 5, 14–16, 38–40], included patients were recruited arbitrarily on the basis of combined KSS of >180 and willingness to provide informed consent to participate in the activities. Fluoroscopic imaging of knee kinematics was obtained during a nonweight bearing kneeling and a weight bearing lunge activities using a standardized technique that has been widely described (Figure 3) [5, 38–40]. For the kneeling activity, the patients kneeled on a padded chair with their operated knee and flexed to their maximum comfortable flexion. For the lunge activity, the subjects placed their foot upon a 30 cm riser and lunged forward with their operated knee to maximum comfortable flexion. Once the subjects had reached their maximal flexed position in each activity, one to three seconds of fluoroscopic images were digitally recorded. The subjects' postures were not constrained in any way during these activities. An investigator was always available to assist the subjects in case of misbalance by holding their hands or forearms. Using the sagittal plane images, three-dimensional knee kinematic was analyzed using a model-image registration technique [38]. Maximum skeletal knee flexion was measured as the angle between the axes of the femoral and tibial shafts. Condylar translations were determined from the anteroposterior location of the lowest point on each femoral condyle relative to the transverse plane of the tibial baseplate, with the coordinate system defining femoral anterior translation as positive and femoral posterior translation as negative values. Standard errors for this fluoroscopic imaging process are approximately 0.5° to 1.0° for rotations and 0.5 to 1.0 mm for translations in the sagittal plane [38].

## 3. Results

Prospectively measured clinical scores and radiographic outcomes were generally good to excellent after five to ten years of in vivo function (Table 1). Among all 159 TKA, KSS knee and function scores at last follow-up averaged 96 ± 7 (range, 55 to 100) and 92 ± 13 (range, 50 to 100), respectively. Stability scores reported within the KSS knee score were perfect (25 points) for 97% and 86% of the TKA in the PCL-retained and PCL-resected groups, respectively. Passive flexion at last follow-up averaged 124° + 9° (range, 90° to 150°), with 126 TKA (79%) exhibiting 120° or more flexion. Small, but statistically significant, differences in knee flexion and function scores were also noted, with the PCL-resected group exhibiting an average of 5° more flexion (*P* = 0.002) but an average function score that was 7 points lower (*P* = 0.003) compared to the PCL-retained group (Table 1). The clinical cohort in each of the PCL-retained and PCL-resected groups had dissimilar patient ages (*P* < 0.001), which were generally representative of the diverse geographic locations of the two clinical sites.

Radiological assessments by a nonauthor third party orthopaedic surgeon were completed on 141 TKA that had sufficient follow-up films for examination. Restoration of suitable limb alignment was noted, recorded as part of the KSS knee score. Narrow (less than 2 mm), nonprogressive radiolucent lines were noted on 19% of the TKA, including 27% and 2% of the TKA in the PCL-retained and PCL-resected groups, respectively. None showed progression on the yearly radiographs. The largest proportion of radiolucent lines were noted in two femoral regions of dense cortical bone on the anterior femur (zone 1) or in sclerotic bone on the posterior femur (zone 4) and one tibial region on the medial tibial pleateau (zone 1). Three TKA in the PCL-retained group exhibited wide radiolucent lines (more than 2 mm) in one zone and were also classified as nonprogressive on serial radiographs. None of the TKA in the PCL-resected group had wide radiolucent lines.

Detailed fluoroscopic analysis revealed few significant differences between the PCL-retained and PCL-resected groups (Table 2). Maximum skeletal flexion during nonweight bearing kneeling ranged from 109° to 160°, with no statistical difference (*P* = 0.15) between the PCL-retained group measuring an average of 131° ± 13° and the PCL-resected group averaging 124° ± 11°. Tibial axial rotation averaged 10° for both groups during kneeling. Lateral femoral rollback in kneeling averaged 10 mm in the PCL-retained group, which was significantly larger (*P* = 0.01) than the average 5 mm in the PCL-resected group. Maximum skeletal flexion for the lunge activity averaged 120° ± 11° and 123° ± 17° for the PCL-retained and PCL-resected groups, respectively. In the PCL-resected group during the lunge, three patients showed slight articular lift-off in the medial compartment at flexion angles greater than 139°, consistent with observations from normal knees in deep flexion [40]. Tibial axial rotation and lateral femoral rollback were statistically similar for both groups, averaging 9° to 11° of rotation and 8 mm to 9 mm of rollback.

## 4. Discussion

Much of the clinical success of TKA is partially dependent upon using TKA designs with suitable congruity and constraint to provide adequate knee joint stability throughout the full range of motion [3–17]. AP stability during TKA is achieved by proper handling of soft tissues, implanting knee prostheses with suitable thickness, proper alignment, and conforming surfaces, helping to restore tension to the remaining ligament structures [20, 25]. However, it remains unclear precisely how much conformity is required for successful TKA [30]. The current study demonstrated that a TKA articular design with progressive congruency in the lateral compartment (Figures 1 and 2) can provide for femoral condyle rollback in maximal flexion activities and good clinical outcomes at midterm follow-up. Positive clinical and functional performance was achieved in patients whose PCL was either meticulously maintained or summarily excised (Table 1), demonstrating the suitability of using this TKA design with either surgical technique.

Physical examinations and in vivo fluoroscopic analysis showed reasonably consistent flexion kinematics in knees with or without a PCL (Tables 1 and 2). Average passive flexion ranged from 122° to 127° for all TKA in the clinical groups and average flexion during the kneeling and lunge activities ranged from 120° to 131°. This maximum flexion measurement is equivalent to or better than flexion reported in American patient populations with contemporary TKA including the asymmetrically constrained high-flexion TKA and posterior-stabilized TKA [16, 18, 21, 24]. Our findings also are very comparable to results previously reported for Japanese patients implanted with the same TKA design (3D Knee) using a PCL-preserving surgical technique [41]. In that study [41], patients achieved 127° ± 13° (range, 115° to 160°) of passive flexion during clinical evaluation, 123° ± 13° (rang, 107° to 156°) during kneeling, and 124° ± 15° (range, 107° to 163°) during squatting.

We evaluated TKA performance using clinical outcome scores, in which stability was assessed using the KSS knee score (25 points are assessed for stability), and functional fluoroscopic assessments during deep flexion activities. This combination was selected because previous studies have been unable to differentiate the performance of different TKA designs using only clinical outcomes or gait analysis, even when comparing vastly different articular geometries such as cruciate-retaining or posterior-stabilized TKA [15, 23, 42–45]. In a large randomized controlled study designed with sufficient statistical power to assess TKA performance in bilateral TKA patients, Kim et al. [23] were unable to detect differences between cruciate-retaining TKA and posterior-stabilized TKA using clinical outcome scores and goniometric measurements of the range of motion. Other studies have used combined clinical outcome scores and fluoroscopic assessment of knee flexion kinematics to detect inferior functional performance of TKA designs [14, 39, 40, 46]. In a randomized controlled study of PCL-retaining mobile-bearing TKA assessed using fluoroscopy, Lützner et al. [46] reported lack of functional improvement in some patients supported by significantly lower KSS function scores and axial rotation biased toward femoral internal rotation during nonweight bearing passive knee flexion. Ploegmakers et al. [39] compared two PCL-retaining TKA designs using fluoroscopy during deep knee flexion and reported inferior functional performance for one design supported by greater anterior condylar translation and corresponding greater joint stiffness and disability WOMAC scores. In a randomized controlled fluoroscopy study, Victor et al. [40] reported more stable function of the femoral medial condyle during a deep flexion lunge in posterior-stabilized TKA compared to cruciate-retaining TKA.

A critical question for TKA designs intended for use with or without the PCL is whether the tibiofemoral articulation provides adequate knee stability to eliminate or reduce unproductive AP sliding during dynamic activities like gait and stair climbing. The larger condylar translations (approximately 10 mm) recorded during the deep flexion activities in the current study are consistent with the TKA achieving femoral rollback essential for accomplishing deep flexion kneeling, lunge, and step up/down activities [39, 40]. The approximately 10° of tibial internal rotation (femoral external rotation) similar to healthy normal knee kinematics is known to aid patellar tracking and enhance the quadriceps function during knee flexion [47]. Similar magnitudes of axial rotation during gait and stair activities have been reported for successful TKA cruciate-retaining and posterior-stabilized designs [14, 15]. The articular constraint provided by the TKA design in the current study provided adequate femoral rollback and intrinsic stability to control tibiofemoral motions during deep flexion activities, which may help to avoid developing compensatory hamstrings cocontraction patterns that can limit functional strength [3].

This new TKA design concept was originally introduced in 2001 and was based on new knowledge from studies of in vivo kinematics and retrieved implant analysis [16]. Based on the current study's midterm clinical and functional outcomes, there were few differences between the PCL-retained and PCL-resected groups. Stability scores recorded within the KSS were perfect for 150 (94%) of these TKA. We conclude that this design is beneficial for addressing the wide range of joint laxity that can occur with patient variation and the PCL-retaining and PCL-resecting surgical techniques. Longer duration clinical follow-up and tracking of the polyethylene wear that occurs with in vivo function are ongoing to fully characterize the longevity of this design as related to the highly congruent lateral femoral condyle and widened medial condyle design features (Figures 1 and 2).

The most widely used design concept for providing AP stability after resection of both cruciate ligaments is to incorporate a femoral cam and tibial post articulation [30]. However, several complications associated with wear of the tibial post and increased strain at the prosthesis-bone interface have emerged with those designs [31, 32, 48–50]. O'Rourke et al. [48] reported osteolysis in 16% of cemented IB-II posterior-stabilized TKA at 5 to 8 years follow-up. Similarly, Mikulak et al. [49] reported osteolysis and a 3% rate of revision for loosening associated with high rotational constraint in TKA with a cam and post articulation. Femoral component loosening associated with weight bearing maximum flexion also has been reported for more modern high-flexion TKA designs having a cam and post articulation, with Han et al. [50] reporting a 38% loosening rate in Legacy PS-Flex TKA at 2 to 4 years follow-up. In the current study, there was a notable absence of progressive radiolucent lines at five to ten years of follow-up, suggesting that the forces developed by articular constraints are well-tolerated throughout the midterm duration. Therefore, the stability provided by the TKA design in the current study, which does not depend on a cam and post mechanism, appears to provide a considerable advantage for avoiding such documented complications with cam and post designs.

Several limitations are recognized. Outcomes were assessed solely using KSS knee and function scores, as was the clinical standard for the involved clinics at the start of this study. KSS are known to be weak predictors of differences between patients. While other clinical outcome measures may have detected outcome differences between the study groups, the current study incorporated quantitative radiographic assessments and functional fluoroscopic analysis to supplement the KSS. Regarding surgical technique, one of the known differences between PCL-retaining and PCL-sacrificing surgical techniques is the potential for an increased flexion gap in the latter case. When noted intraoperatively, this was accommodated through the use of thicker tibial inserts in the latter case. The proportion of TKA having nonprogressive radiolucent lines was greater for the PCL-retained group than for the PCL-resected group. This is likely due to technicians who were more demanding in aligning the beam with the prostheses and newer radiography equipment in use at the clinic where the PCL-retained group were followed up. These factors combined to produce better views of the interface between the bone and cement on higher resolution images, aiding detection and follow-up of any progression of observed radiolucent lines at that clinical site.

Limitations related to the limited size of patient cohorts are noted. Dissimilar surgical volume at the two involved clinical sites led to unequally sized patient cohorts with suitable follow-up data acquired within the timeframe of this study. While such dissimilarity can lead to overly broad population variance, and contributed to the differences in patient ages for each group, the authors chose to err on inclusion rather than reduce the PCL-retaining group from 116 TKA down to 43 TKA. The follow-up rate was less than 75% because of the decision to capture an initial group of 150 TKA in patients who met the inclusion criteria and were willing to come to the clinic for follow-up, rather than trying to follow up all patients, many of whom maintain only seasonal residency in the geographic regions surrounding the clinical sites. Studies involving a sequential series of TKA patients operated with this TKA are needed to fully characterize outcomes from patients who are not motivated to return for follow-up or have reduced willingness to participate in clinical studies. The fluoroscopic analysis included only 33 patients who all had high outcome scores. Such selection criteria have been used in many previously published fluoroscopic studies and have proven to yield significant results [4, 5, 14–16, 38–40].

## 5. Conclusion

This novel TKA design showed excellent functional performance with or without the posterior cruciate ligament over the intermediate time period of five to ten years. The standard implantation technique provided surgeons with the option of using one implant solution when dealing with variable posterior cruciate ligament integrity. The 3D Knee provided the required stability to achieve excellent clinical and radiographic outcomes and consistent kinematic results in both the PCL-retained and PCL-resected patient cohorts. Continued multicentered studies of this TKA design will be necessary to produce the evidence-based data to ultimately prove long-term clinical success.

## Figures and Tables

**Table 1:** Patient demographics and outcome measures for all TKA and for the PCL-retained and PCL-resected groups.

	All TKA	PCL-retained	PCL-resected	P*
Patients	159	116	43	
Sex (F/M, % female)	98/61 (62%)	65/51 (56%)	33/10 (77%)	
Age at index surgery (yrs.)	70 ± 9	72 ± 7	64 ± 9	<0.001
Age at last follow-up (yrs.)	76 ± 8	78 ± 7	71 ± 9	<0.001
KSS (knee)	96 ± 7	96 ± 7	96 ± 5	0.76
KSS (function)	92 ± 13	94 ± 12	87 ± 14	0.003
Maximum knee flexion (°)	124 ± 9	122 ± 9	127 ± 9	0.002

*Significant differences between the PCL-retained and PCL-resected groups were assessed using a Student's *t*-test.

**Table 2:** Kinematics during the kneeling and lunge activities for the PCL-retained and PCL-resected groups (mean ± standard deviation, range).

	Nonweight bearing kneeling			Weight bearing lunge		
	PCL-retained	PCL-resected	P*	PCL-retained	PCL-resected	P*
Patients	20	13		20	10	
Skeletal knee flexion (°)	131 ± 13 (109 to 160)	124 ± 11 (105 to 141)	0.15	120 ± 11 (95 to 147)	123 ± 17 (87 to 145)	0.54
Implant valgus (°)	−1 ± 2 (−3 to 2)	1 ± 3 (−3 to 8)	0.02	−1 ± 1 (−2 to 1)	1 ± 2 (−5 to −1)	0.00
Tibial external rotation (°)	−10 ± 4 (−18 to −3)	−10 ± 6 (−19 to −1)	0.75	−11 ± 4 (−16 to −3)	−9 ± 4 (−18 to −4)	0.25
Medial condyle AP (mm)	−2 ± 4 (−10 to 9)	2 ± 4 (−3 to 9)	0.02	0 ± 4 (−6 to 8)	−2 ± 4 (−9 to 3)	0.22
Lateral Condyle AP (mm)	−10 ± 4 (−20 to −1)	−5 ± 4 (−9 to 1)	0.01	−8 ± 4 (−15 to −1)	−9 ± 3 (−15 to −5)	0.41

*Significant differences between the PCL-retained and PCL-resected groups were assessed using a Student's *t*-test.
